# Atypical Cutaneous Presentation and Diagnostic Challenges in Advanced Metastatic Testicular Cancer

**DOI:** 10.7759/cureus.65072

**Published:** 2024-07-22

**Authors:** Devaun M Reid, Britannia O Noel, Abraham A Mascio, Dwight Smith, Martin Giangreco

**Affiliations:** 1 Internal Medicine, University of South Florida Morsani College of Medicine, Tampa, USA

**Keywords:** metastatic testicular cancer, dermatology, cutaneous presentation, non-seminoma germ cell tumor, testicular cancer

## Abstract

A 29-year-old male presented with acute left-sided weakness in both the upper extremity (UE) and lower extremity (LE), an atypical symptom for testicular cancer but not uncommon for brain metastasis. Testicular cancer usually manifests as a testicular mass or discomfort. His medical history included a previously resected testicular mass, with pathology results unknown due to the patient being lost to follow-up. Upon examination, he exhibited significant neurological deficits and multiple subcutaneous nodules. Imaging revealed multiple enhancing brain lesions and widespread metastases to the lungs and other regions. Laboratory tests showed elevated alpha-fetoprotein and lactate dehydrogenase levels, supporting a diagnosis of advanced non-seminomatous germ cell tumor. He received multidisciplinary treatment, including dexamethasone, levetiracetam, and chemotherapy. The patient responded well to the treatment, showing significant improvement in neurological function and stabilization of his condition. This case underscores the diagnostic and therapeutic challenges of metastatic testicular cancer, particularly with rare presentations such as cutaneous involvement, and highlights the importance of comprehensive diagnostic evaluations and multidisciplinary care.

## Introduction

Testicular cancer predominantly affects younger males and frequently metastasizes to the retroperitoneal lymph nodes and distant organs such as the lungs and liver. Rarely, cutaneous metastases (spread of cancer to the skin) appear as an initial manifestation, posing significant diagnostic and therapeutic challenges. Metastasis typically occurs via lymphatic and hematogenous (blood) routes. Effective management of metastatic testicular cancer, including non-seminomatous germ cell tumors (a more aggressive type of testicular cancer), involves a multidisciplinary approach, including radical inguinal orchiectomy to remove the primary tumor, followed by chemotherapy, commonly using the bleomycin, etoposide, and cisplatin (BEP) regimen, which is highly effective for advanced disease. Retroperitoneal lymph node dissection (RPLND) is performed to address nodal metastasis, particularly in cases resistant to chemotherapy [1].

Radiation therapy is selectively utilized, especially for seminomas, which are highly radiosensitive, to target metastatic lymph nodes. Emerging targeted therapies and immunotherapies are being investigated to address specific genetic mutations and immune evasion mechanisms in testicular cancer cells. Regular follow-up with imaging and tumor marker assessments of alpha-fetoprotein (AFP), human chorionic gonadotropin (HCG), and lactate dehydrogenase (LDH) are crucial for early detection of recurrence and management of long-term treatment sequelae. Clinical trials offer access to innovative treatments, contributing to improved outcomes and understanding of the disease. Through comprehensive and evolving intervention strategies, the goal is to enhance survival rates and quality of life for patients with metastatic testicular cancer.

## Case presentation

We present the case of a 29-year-old male with a history of testicular germ cell tumor with metastasis. He presented with a one-day history of left leg and arm weakness. He reported waking up unable to lift his left arm or leg off the bed, despite having full function previously and actively working in his lawn care business. His history included a testicular mass, which was resected in March 2014. However, he was lost to follow-up and unaware of the pathology results. Upon presentation, he had growing nodules on his anterior scalp, right buttock, and thigh, as well as pain from subcutaneous nodules.

Upon physical examination, he was alert and oriented with vital signs of 96°F, HR 80 beats per minute, respiratory rate18 breaths per minute, and blood pressure 109/61 mmHg. The physical exam revealed that the pupils were equal and reactive, had a regular heart rate, clear lungs, and a soft, non-tender abdomen. There were ~4 cm subcutaneous masses on the right thigh and buttock, strength was 2/5 in the left arm and leg, deep tendon reflexes (DTRs) were 1/4 on the left, and the scalp had erythematous, crusted nodules (~2×2 cm). He was in no acute distress. Additionally, he was negative for murmurs, wheezes, rales, rebound, guarding, hepatosplenomegaly, edema, or sensory deficits.

**Figure 1:**
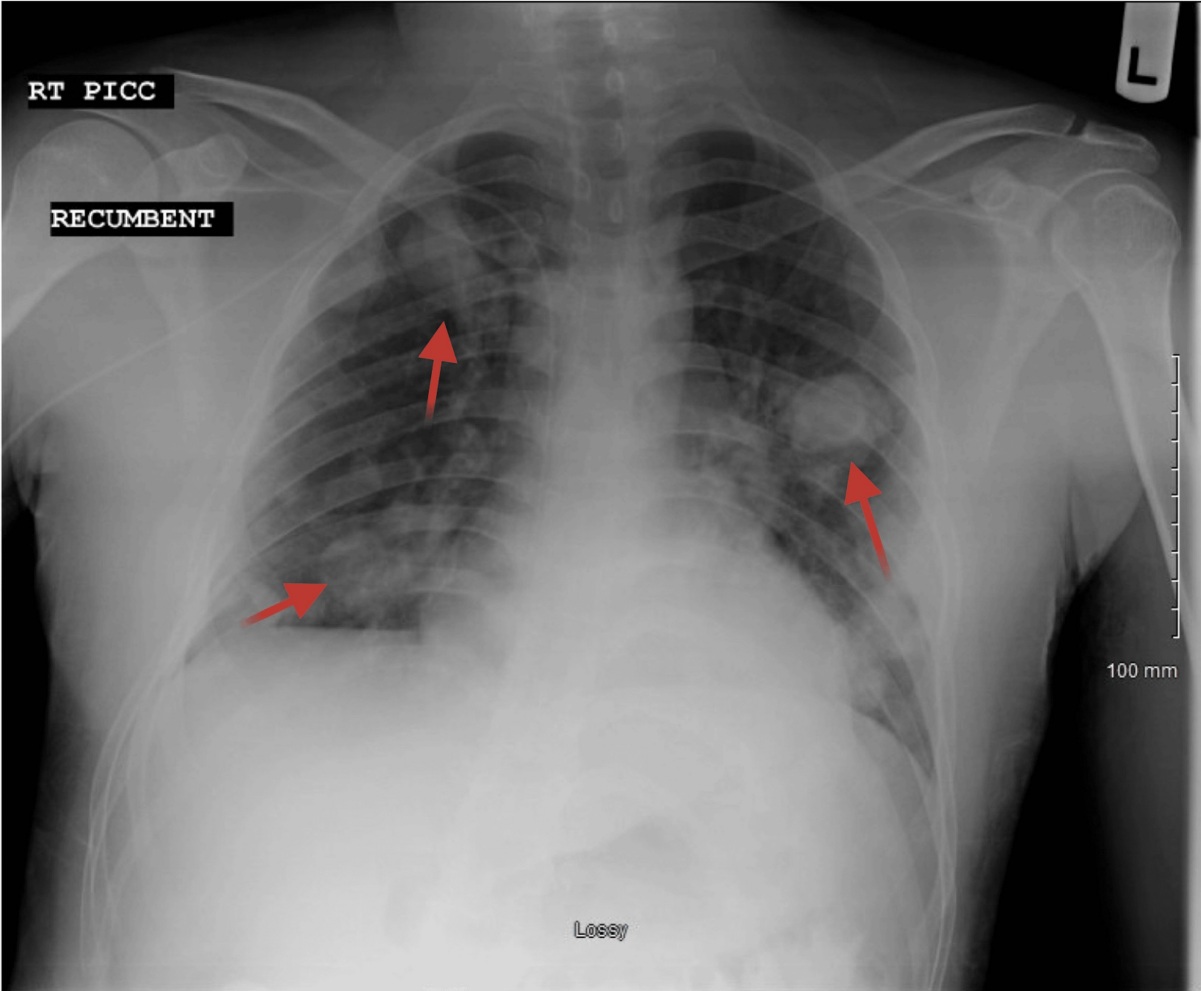
X-ray of the chest anteroposterior portable - multiple masses in both lungs

Initial imaging studies included an MRI of the brain, which showed multiple intra-axial heterogeneous enhancing lesions in the right frontal, parietal, and occipital lobes, with the largest lesion measuring 3.5 × 3.0 × 3.4 cm. A chest X-ray further confirmed multiple masses in both lungs, up to 3.5 cm (Figure 1).

Laboratory tests revealed elevated AFP at 2178 ng/mL, LDH at 1088 U/L (previously 1417 U/L), and negative HCG. A skin biopsy of the scalp nodules showed malignancy consistent with metastatic testicular cancer. During the initial hospitalization, neurosurgery was consulted due to the brain lesions, and he was placed on dexamethasone 4 mg every 8 hours. Hematology/oncology started him on cisplatin and etoposide. The patient showed significant improvement in his left-sided weakness and was able to walk with crutches. He was discharged in improved condition with instructions for follow-up treatments, including a scheduled return to the infusion center for bleomycin.

Six months after his initial diagnosis of brain lesions, the patient was readmitted for routine chemotherapy. His condition had relapsed, progressing to metastatic testicular cancer with brain involvement and seizures. Upon readmission, he reported awakening unable to lift his left arm or leg off the bed, a stark contrast to his previous full function while working in his lawn care business. He was being treated with dexamethasone 4 mg every 8 hours for cerebral edema and levetiracetam 500 mg twice daily for seizure management. Hematology/oncology initiated paclitaxel, ifosfamide, and cisplatin (TIP) chemotherapy: paclitaxel 250 mg/m² IV over 24 hours on Day 1, ifosfamide 1.5 g/m² IV on Days 2-5, cisplatin 25 mg/m² IV on Days 2-5, and mesna 500 mg/m² IV before ifosfamide and at 4 and 8 hours after ifosfamide daily on Days 2-5. He tolerated the treatment well, experiencing no significant issues during the hospital stay. Upon discharge on March 29, he was prescribed filgrastim 5 mcg/kg subcutaneously on Days 7-18, continued on dexamethasone and levetiracetam, and scheduled for follow-up with hematology/oncology. Currently, the patient is stable, following his chemotherapy regimen, and under close monitoring by his oncology team.

## Discussion

This case illustrates several critical teaching points. Testicular cancer, especially germ cell tumors, typically presents in younger males and is highly treatable when diagnosed early [2]. However, metastatic presentations, such as in this case, pose significant challenges and highlight the importance of comprehensive diagnostic approaches [3]. Brain metastases and skin lesions in metastatic testicular cancer present clinical challenges rooted in the disease’s typical patterns of metastasis. Testicular cancers, predominantly germ cell tumors, commonly metastasize via the lymphatic system to regional lymph nodes, lungs, and liver. The rarity of brain metastases is attributed to the blood-brain barrier, which limits the entry of circulating tumor cells into the central nervous system. When brain metastases do occur, they often signify the hematogenous spread of highly aggressive cancer cells, highlighting advanced disease progression [3].

Skin metastases in testicular cancer are even less frequent, largely due to the skin’s anatomical and physiological barriers against metastatic colonization. The dense extracellular matrix and robust immune surveillance within the skin make it an uncommon site for secondary tumor deposits. The occurrence of both brain and skin metastases in a patient with testicular cancer thus indicates an exceptional and atypical disease course, suggesting extensive systemic dissemination and a particularly aggressive tumor phenotype [4].

Moreover, the patient’s initial presentation with neurological deficits (left arm and leg weakness) is unusual for testicular cancer, which typically presents with a testicular mass or discomfort [4]. The neurological symptoms prompted imaging that revealed multiple brain lesions, a finding more commonly associated with primary brain tumors or other cancers such as lung cancer [5]. The discovery of multiple enhancing brain lesions with surrounding edema, without midline shift, highlighted the need for a broad differential diagnosis and multidisciplinary consultation, including neurosurgery and oncology [6].

The rapid progression from a testicular mass to multiple metastases, including brain, lung, and subcutaneous tissues, is noteworthy. Metastatic testicular cancer can present with distant metastases at the time of diagnosis in about 15-20% of cases, but brain metastases are relatively rare, occurring in only 1-2% of patients [7]. This rarity underscores the significance of thorough staging and imaging studies, including MRI and CT scans, in patients presenting with atypical symptoms [8]. The patient’s history of being lost to follow-up after initial surgery for a testicular mass is a critical point. Pathology from the initial surgery, which showed teratoma, seminoma, and yolk sac elements, was crucial for diagnosing the mixed germ cell tumor. This emphasizes the importance of follow-up in oncological care to ensure timely treatment and prevent progression [9].

Laboratory findings, particularly elevated AFP and LDH levels, were consistent with the diagnosis of non-seminomatous germ cell tumors. AFP is a key tumor marker in non-seminomatous germ cell tumors and correlates with disease burden and response to therapy [10]. The normal HCG level helped differentiate the tumor type, as seminomas typically elevate HCG but not AFP [11]. The management of this case involved a combination of chemotherapy regimens, including bleomycin, etoposide, and cisplatin (BEP), and later TIP, reflecting standard treatment protocols for advanced germ cell tumors [12]. The use of dexamethasone to manage neurological symptoms and Keppra for seizure prophylaxis were appropriate given the brain metastases. The improvement in neurological function following initial treatment and the patient’s stabilization post-chemotherapy further highlights the effectiveness of aggressive multimodal therapy in advanced testicular cancer.

## Conclusions

In conclusion, the occurrence of brain metastases and skin lesions in metastatic testicular cancer underscores the formidable challenges posed by its atypical patterns of spread. While testicular cancers typically metastasize via the lymphatic system to predictable sites such as regional lymph nodes, lungs, and liver, the rarity of brain metastases can be attributed to the formidable blood-brain barrier. The infrequency of skin metastases further highlights the skin's effective barriers against metastatic colonization. When brain and skin metastases do manifest, they signal an advanced disease state characterized by aggressive tumor biology and extensive systemic dissemination. Managing such cases requires a comprehensive, multidisciplinary approach that integrates advanced imaging, precise staging, and targeted therapeutic strategies to optimize patient outcomes in the face of these clinical and medical complexities.
